# Indications and Outcomes for Arthroscopic Hip Labral Reconstruction With Autografts: A Systematic Review

**DOI:** 10.3389/fsurg.2020.00061

**Published:** 2020-10-16

**Authors:** Felipe S. Bessa, Brady T. Williams, Evan M. Polce, Mansueto Neto, Flávio L. Garcia, Gustavo Leporace, Leonardo Metsavaht, Jorge Chahla

**Affiliations:** ^1^Instituto Brasil de Tecnologias da Saúde (IBTS), Rio de Janeiro, Brazil; ^2^Division of Young Adult Hip Surgery, Department of Orthopaedic Surgery, Rush University Medical Center, Chicago, IL, United States; ^3^Physioterapy Research Group, Bahia Federal University, Salvador, Brazil; ^4^Ribeirão Preto Medical School, Ribeirão Preto, Brazil; ^5^Imaging Diagnostic Department, São Paulo Federal University, São Paulo, Brazil

**Keywords:** suction seal, hip arthroscopy, autograft, femoroacetabular impingement syndrome, labrum

## Abstract

**Background:** The acetabular labrum plays a major role in hip function and stability. The gold standard treatment for labral tears is labral repair, but in cases where tissue is not amenable to repair, reconstruction has been demonstrated to provide superior outcomes compared to debridement. Many types of grafts have been used for reconstruction with good to excellent outcomes. Autograft options include iliotibial band (ITB), semitendinosus, and indirect head of the rectus femoris tendon, while allografts have included fascia lata and gracilis tendon allografts.

**Questions/Purposes:** As allografts are not always readily available and have some inherent disadvantages, the aims of this systematic review were to assess (1) indications for labral reconstruction and (2) summarize outcomes, complications, and reoperation rates after arthroscopic labral reconstruction with autografts.

**Methods:** A systematic review of the literature was performed using six databases (PubMed, CINAHL, Cochrane Central Register of Controlled Trials, Cochrane Database of Systematic Reviews, Scopus, and Google Scholar) to identify studies reporting outcomes for arthroscopic labral reconstruction utilizing autografts, with a minimum follow-up of 1 year. Study design, patient demographics, autograft choice, complications, donor site morbidity, reoperation rates, conversion to arthroplasty, and patient reported outcomes were extracted and reported.

**Results:** Seven studies were identified for inclusion with a total of 402 patients (173 females, age range 16–72, follow-up range 12–120 months). The most commonly reported functional outcome score was the modified Harris Hip Score (mHHS), which was reported in six of seven studies. Preoperative mHHS ranged from 56 to 67.3 and improved postoperatively to a range of 81.4–97.8. Conversion to total hip arthroplasty and reoperation rates ranged from 0 to 13.2% and 0 to 11%, respectively. The most common indication for labral reconstruction was an irreparable labrum. Autografts utilized included ITB, hamstring tendons, indirect head of rectus femoris, and capsular tissue.

**Conclusions:** Arthroscopic autograft reconstruction of the acetabular labrum results in significant improvement in the short- and mid-term patient reported outcomes, for properly selected patients presenting with pain and functional limitation in the hip due to an irreparable labral injury.

## Introduction

In the last quarter of century, much has been learned regarding the management of acetabular labral injuries (1). Historically, labral tears were treated with debridement or excision (2). However, improved understanding of the importance and function of the labrum as a hip stabilizer and its suction seal effect (3–5) has led to development of labral repair techniques. Repairs are typically performed with the use of suture anchors, and have quickly revolutionized the treatment of labral tears, demonstrating improved outcomes compared to debridement or resection (6–9).

In addition to repair, reconstruction techniques have also been developed in order to treat patients with significant labral tears or insufficient labral tissue not amenable to repair (10–15). Outcomes following reconstruction have also demonstrated significant improvements in patient reported pain and function in clinical studies (11–22). Mechanistically, reconstruction of the labrum has demonstrated an ability to, at least in part, restore the stability of the suction seal effect as shown in *in vitro* studies (3–5).

The overall improvements in outcomes have been since summarized in recent systematic reviews (11, 13, 23). However, prior systematic reviews have included studies with significant heterogeneity regarding the type and source of graft tissue utilized in the procedure, including both auto- and allografts, and the technique, including both open and arthroscopic reconstructions. This potentially clouds comparisons that may not be truly reflective of the current outcomes data following modern techniques.

Hip arthroscopy represents the modern and preferred method for labral reconstruction, with arthroscopic procedures constituting 86% of these procedures (24). Arthroscopic procedures result in superior outcomes, lower reoperation rates (25), and expedited recovery compared to surgical dislocation of the hip (26). Despite inherent advantages of allografts, such as decreased surgical time and avoidance of donor-site morbidity, some disadvantages should be taken into account such as potential disease transmission, delayed incorporation, increased costs and patient refusal, making autografts the preferred source for a subset of surgeons (10, 23, 27–29). Allografts may also be less readily available, or non-existent options for surgeons in certain parts of the world. Therefore, the purpose of this study was to systematically review the reported indications for labral reconstruction, and to assess the outcomes, complications and reoperations after arthroscopic labral reconstructions with the exclusive utilization of autografts.

## Materials and Methods

This systematic review of the literature was performed according to the Preferred Reporting Items for Systematic Reviews and Meta-Analyses (PRISMA) guidelines (30). Potential studies were identified by searching the following sources: PubMed, CINAHL (Cumulative Index of Nursing and Allied Health Literature), Cochrane Central Register of Controlled Trials, Cochrane Database of Systematic Reviews, Scopus and Google Scholar. Searches were performed by one reviewer, with the support of a medical librarian. The utilized terms of the search were “labral” or “labrum,” “reconstruct^*^,” “arthroscop^*^,” and “hip.” The search strategy for PubMed is summarized in Table 1. Eligible articles included longitudinal studies that reported outcomes following arthroscopic labral reconstruction with autografts, with a minimum of 1 year of follow-up. Studies were excluded if they did not report postoperative outcomes, utilized allografts or described open reconstruction. Technical notes, review articles, systematic reviews, animal and *in vitro* studies were also excluded.

**Table 1:** PubMed search strategy.

(Labral[title/abstract] OR labrum[title/abstract])
and (reconstruct*[title/abstract])
and (“Arthroscopy”[Mesh] OR arthroscop*[title/abstract])
and (“Hip Joint/surgery”[Mesh] OR hip[title/abstract])

The list of titles and abstracts from each database was independently evaluated by two reviewers (FSB and FLG) to identify potential studies for the systematic review. If at least one author deemed a study eligible, the full text was obtained for a complete assessment. Full texts of selected studies were independently assessed for inclusion or exclusion criteria. Disagreements were discussed by the authors, and a final decision was reached by consensus. References from each identified article were reviewed to identify other potentially eligible studies.

Two reviewers (FSB and FLG) then independently extracted the data from published studies using standard data extraction forms adapted from the Cochrane Collaboration (31) model including: (1) demographics of the study population, such as gender and mean age; (2) details of the arthroscopic technique for autograft harvest and labral reconstruction; (3) follow-up duration; (4) patients lost to follow-up or rates of withdrawal; (5) outcome measures (patient reported outcomes, reoperation rates, donor site morbidity, and conversion to arthroplasty) and (6) study results and conclusions.

Two independent reviewers (FSB and FLG) assessed the quality of the included studies according to the Methodological Index for Non-randomized Studies (MINORS) (32). This is a validated instrument designed to assess the methodological quality of non-randomized studies, whether comparative or non-comparative, using 12 items, with four of them exclusively applying to comparative studies. Each item is scored “0” if not reported, “1” if inadequately reported or “2” if adequately reported. Non-comparative studies have a maximum score of 16 and comparative studies have a maximum score of 24. Any disagreement between the two reviewers regarding any item score was resolved by consensus.

Continuous data is presented as mean ± standard deviation (range) unless otherwise stated. A meta-analysis for this systematic review was not appropriate due to lack of randomized comparisons, variable patient reported outcomes (PROs) used in the studies, small sample sizes, and differences in surgical techniques between the studies. Therefore, the authors avoided the inappropriate pooling or comparison of data that may potentially lead to inaccurate conclusions. Therefore, data from the included studies was qualitatively synthesized and presented in narrative and tabular formats. Forest plots were constructed to depict outcomes reported in a minimum of three studies, including improvement in PRO scores (Modified Harris Hip Score, mHHS; Hip Outcome Scale - Sports Subscale, HOS-SS; Non-Arthritic Hip Score, NAHS) and proportion of revisions and conversions to total hip arthoplasty (THA). Heterogeneity was assessed with *I*-squared (*I*^2^) tests. All studies used a *p*-value of <0.05 to denote statistical significance. Statistical analysis was performed using the computing software R (R version 1.2.1335, R Foundation for Statistical Computing, Vienna, Austria).

## Results

**Figure 1:**
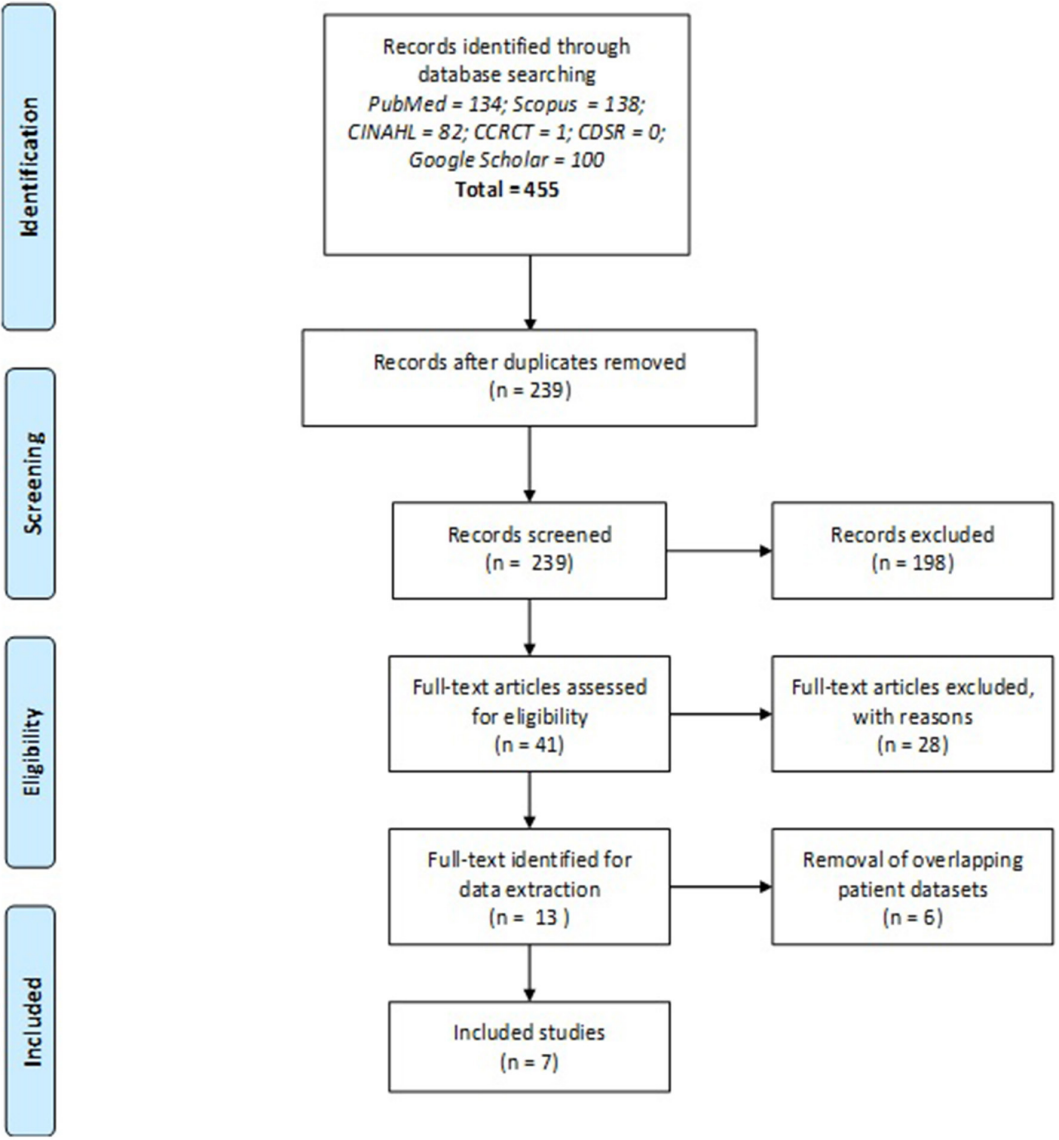
PRISMA 2009 Flow Diagram (30). CINAHL, Cumulative Index of Nursing and Allied Health Literature; CCRCT, Cochrane Central Register of Controlled Trials; CDSR, Cochrane Database of Systematic Reviews.

Query of the 6 online databases yielded 455 candidate results. After exclusion of 216 duplicate studies, 239 titles and abstracts were screened. After the initial screening, 41 full text articles were assessed, from which 28 were excluded after application of the inclusion and exclusion criteria. From the remaining 13 studies, 6 were excluded for presenting data from overlapping patient samples included elsewhere in the review. In such instances, the study with the largest patient sample (317 patients) was selected for inclusion. A total of seven studies were included in the final systematic review (15–21). A cross-reference of bibliographies of the included studies did not yield any additional studies for inclusion. The flow diagram according to PRISMA is presented in Figure 1.

A total of 7 studies and 402 patients (229 males, 173 females) were included in this systematic review. Three studies were retrospective cohort studies (level of evidence III) (18, 19, 21) and four studies were retrospective case series (level of evidence IV) (15–17, 20). All patients underwent arthroscopic labral reconstruction with autografts; however, graft choices were variable across studies. The grafts utilized included iliotibial band (ITB) in two studies (342 patients) (15, 19), indirect head of rectus femoris in three studies (32 patients) (16, 17, 20), gracilis tendon in one study (8 patients) (18), semitendinosus tendon in one study (12 patients) (21), and capsule tissue in one study (8 patients) (20). All studies had a minimum length of follow-up of 12 months (range, 12–120 months). The indications for reconstruction were disrupted labral seal, labral tears that were not amenable to repair (including deficient, degenerative, damaged, surgically debrided, calcified, flattened, frayed or non-viable labrum) and failed prior surgical repair. MINORS scores ranged from 10 to 12 in non-comparative studies, and 15 to 19 in comparative studies. Demographics of the included studies are summarized in Table 2.

**Table 2:** Demographics of studies included in this Systematic Review.

**Study**	**Type of study**	**Number of patients** **(male/female)**	**Age (years)** **Mean (range)**	**Follow-up (months)**	**MINORS Score (Ideal score)**	**Indications for reconstruction**
Lebus et al. (15)	Retrospective case series	317 (173/144)	33.8 (16–69)^a^ 31.9 (15–64)^b^ 46.2 (20–71)^c^	Minimum 24	10 (16)	Disruption of labral seal with the femoral head
Amar et al. (16)	Retrospective case series	22 (13/9)	43 (22–68)	36.2 (24–72)	12 (16)	Irreparable labrum
Rathi and Mazek (17)	Retrospective case series	7 (5/2)	35 (25–41)	15 (12–18)	12 (16)	Irreparable labrum
Matsuda and Burchette (18)	Retrospective cohort	8 (7/1)	34.6 (18–58)	30 (24–37)	15 (24)	Non-salvageable labrum: severe deficiency in quantity (e.g. segmental loss) and/or quality (e.g. labral ossification)
Nakashima et al. (19)	Retrospective cohort	25 (18/7)	52.6 (20–76)	Minimum 24	19 (24)	Irreparable labrum: severe degenerative frayed labrum, calcified (ossification) labrum, or flattened labrum in patients with healthy cartilage
Locks et al. (20)	Retrospective case series	11 (6/5)	35 (20–51)	65 (12–120)	12 (16)	Absent, severely deficient or irreparable labrum
Maldonado et al. (21)	Retrospective cohort	12 (7/5)	34.8 (17.9–49.9)	Minimum 24	19 (24)	Segmental labral defects and/or non-viable labrum

a *patients without reoperation*.

b *patients with subsequent arthroscopic revision*.

c *patients converted to THA*.

Preoperatively, all studies, except one (18), reported lateral center edge angle (LCEA) and alpha-angle measurement with X-rays. Four studies utilized the Tönnis classification ≥2 as a contraindication for the procedure (16, 18, 19, 21), while two studies used a joint space width <2 mm as a contraindication for reconstruction (15, 16). Four studies reported the use of magnetic resonance imaging (MRI) or arthrography (MRA) preoperatively to confirm the diagnosis of labral pathology (16, 17, 19, 21).

Intraoperatively, all studies reported debridement of the labrum to stable margins before graft insertion. Two studies reported debridement of chondral defects (16, 20), while one study reported microfracture for the treatment of chondral damage (19).

Postoperatively, five studies recommended partial weight bearing (15–18, 21) with varying durations (range, 2–6 weeks), while two studies did not describe a specific rehabilitation protocol (19, 20).

Included studies utilized a variety of PROs. Hip Outcome Score—Activities of Daily Living (HOS-ADL) was used by two studies (15, 20), Hip Outcome Score—Sport Subscale (HOS-SS) by three studies (15, 20, 21), modified Harris Hip Score (mHHS) by six studies (15–17, 19–21), Non-Arthritic Hip Score (NAHS) by three studies (18, 19, 21), Western Ontario and McMaster Universities Osteoarthritis Index (WOMAC) by one study (15), 12-Item Short Form Health Survey (SF-12) physical component summary (PCS) and mental component summary (MCS) by one study (15), International Hip Outcome Tool−12 (iHOT-12) by one study (21) and Visual Analog Scale (VAS) for pain by one study (21).

**Figure 2:**
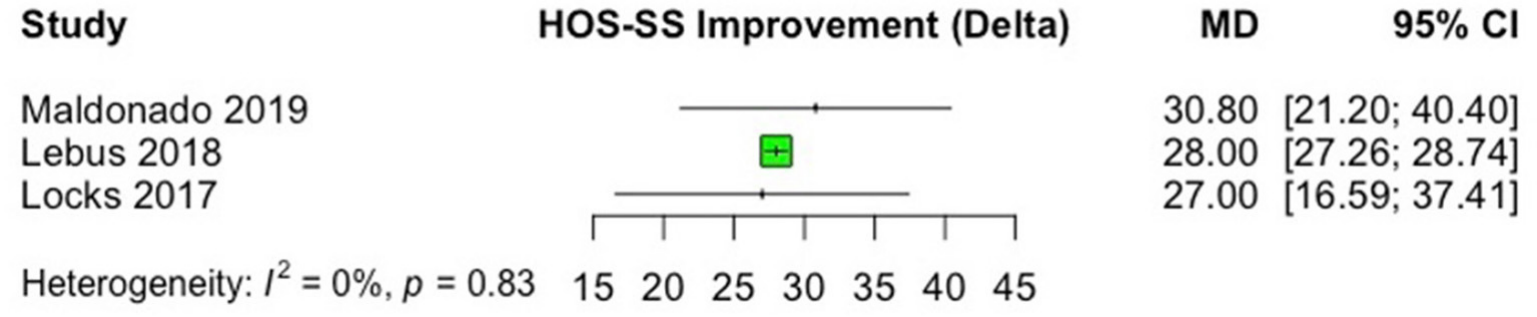
Forest plot displaying the mean preoperative to postoperative improvement (delta, Δ) for the Hip Outcome Score—Sport Subscale (HOS-SS). On the graph, the small vertical lines indicate the mean difference (MD) between preoperative and postoperative HOS-SS scores for each study, with the horizontal lines representing the 95% confidence intervals (CI). The size of the green square is proportional to the relative sample size of each study.

**Figure 3:**
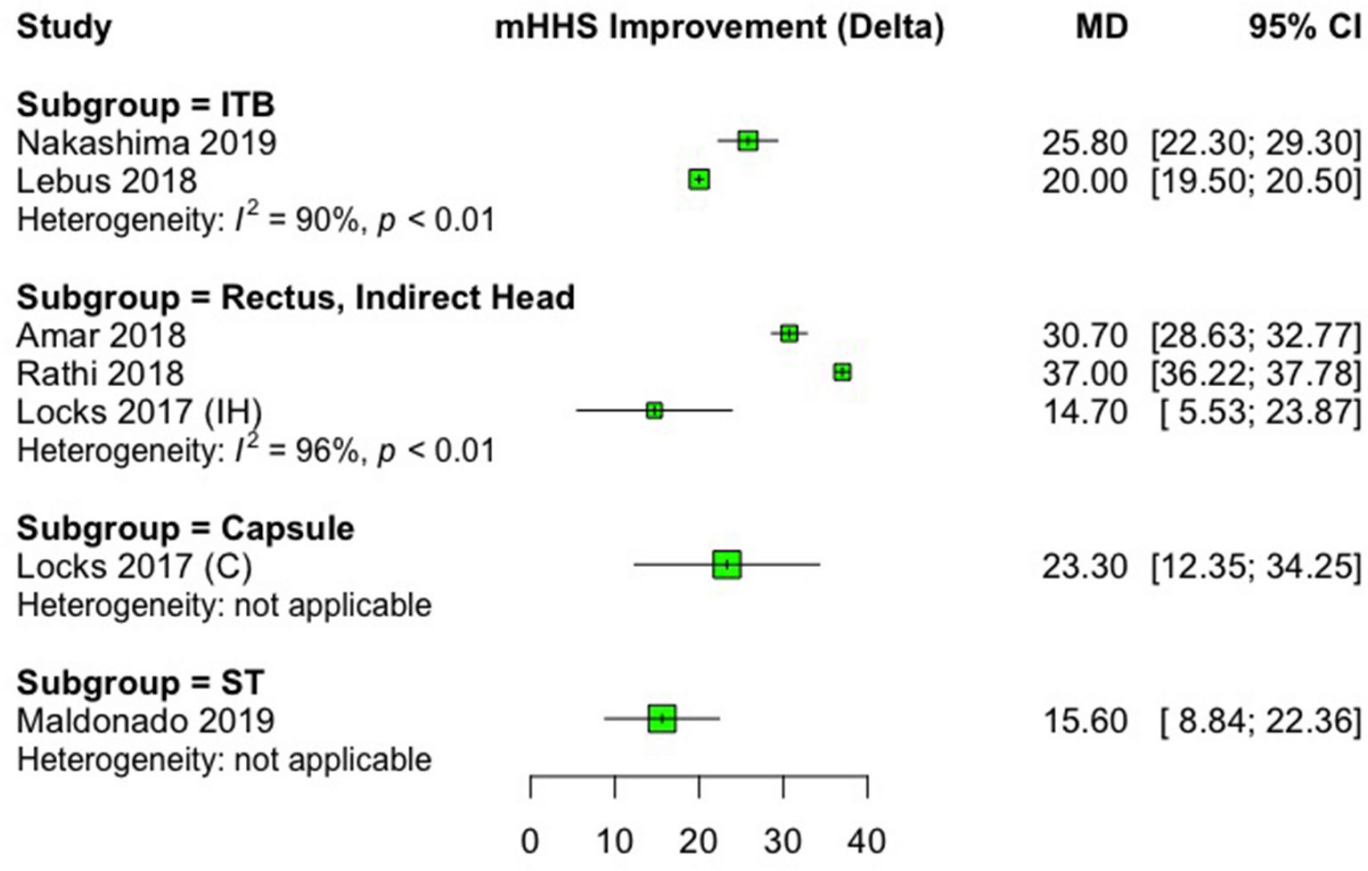
Forest plot displaying the mean preoperative to postoperative improvement (delta, Δ) for the modified Harris Hip Score (mHHS). Each subgroup heading indicates the autograft type that was used. On the graph, the small vertical lines indicate the mean difference (MD) between preoperative and postoperative mHHS for each study, with the horizontal lines representing the 95% confidence intervals (CI). The size of the green square is proportional to the relative sample size of each study. ITB, iliotibial band; ST, semitendinosus tendon.

**Figure 4:**
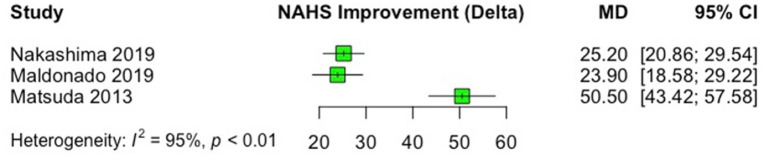
Forest plot displaying the mean preoperative to postoperative improvement (delta, Δ) for the Non-Arthritic Hip Score (NAHS). On the graph, the small vertical lines indicate the mean difference (MD) between preoperative and postoperative NAHS scores for each study, with the horizontal lines representing the 95% confidence intervals (CI). The size of the green square is proportional to the relative sample size of each study.

In all studies, significant improvements were observed from pre- to postoperative scores after labral reconstruction with different types of autografts. Regarding the studies that used the mHHS, the most commonly reported PRO, the minimal clinically important difference (MCID) of +8 points and patient acceptable symptomatic state (PASS) of 74 absolute points (33, 34) was reached in all studies. The only study that did not use mHHS (18), in which NAHS was the PRO of choice, a significant improvement (*p* = 0.008) from a mean preoperative score of 41.9 to mean postoperative score of 91.2 was observed, after labral reconstruction with gracilis tendon autograft. Improvements in PROs that were for at least three studies (HOS-SS, mHHS, and NAHS) are presented as forest plots in Figures 2–4. Significant heterogeneity was observed between studies for mHHS (*I*^2^ = 100%, overall; 96%, rectus, indirect head subgroup; 90%, iliotibial band subgroup) and NAHS (*I*^2^ = 95%), whereas the HOS-SS demonstrated no statistical heterogeneity (*I*^2^ = 0%).

Reporting of autograft donor site morbidity following harvest was variable among included studies. One study reported a mean of 2.4 weeks (range: 1–3 weeks) of incisional knee pain following gracilis tendon harvest without residual weakness or pain (18). Three studies explicitly stated that no complications were observed (17, 20, 21), while three studies did not report the occurrence nor confirm the absence of donor site morbidity (15, 16, 19).

**Figure 5:**
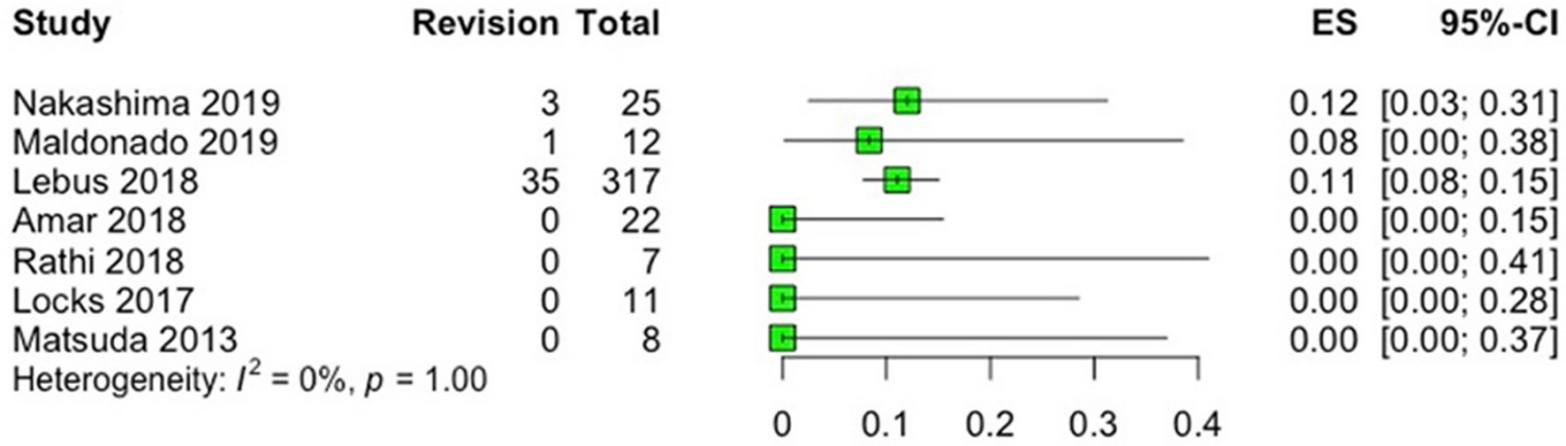
Forest plot displaying the rate of revision arthroscopy (ES; number of revisions/total number of patients) in each study after labral reconstruction. On the graph, the small vertical lines indicate the rate of revision for each study, with the horizontal lines representing the 95% confidence intervals (CI).

**Figure 6:**
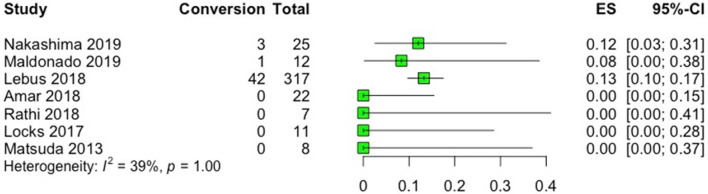
Forest plot displaying the rate (ES; number of conversions/total number of patients) of conversion to total hip arthroplasty (THA) in each study after labral reconstruction. On the graph, the small vertical lines indicate the rate of conversion to THA for each study, with the horizontal lines representing the 95% confidence intervals (CI).

With regards to reoperations, three studies reported rates of revision arthroscopy and conversion to total hip arthroplasty (THA), with ranges of 11–12% and 8.3–13.2%, respectively (15, 19, 21), while four studies reported no revisions or conversion to THA during the follow-up period (16–18, 20) (Figures 5, 6). Of note, no revisions or conversions to THA were observed in the studies that utilized local grafts (indirect head of rectus femoris and capsule tissue) in the follow-up period. Heterogeneity among the included studies was low (*I*^2^ = 41%) and absent (*I*^2^ = 0%) for the outcomes of revision and conversion to THA, respectively. In studies reporting reoperations after labral reconstruction (15, 19, 21), only one (15) reported the indications for revision arthroscopy which were described to be adhesions alone or in combination with labral tear, psoas entrapment, iliopsoas tendinopathy, residual impingement or capsular laxity, trochanteric bursitis, or the need for periacetabular osteotomy. The same study identified prior surgeries and female gender as risk factors for future revision, and older age, higher body mass index (BMI), and decreased joint space (≤2mm) as risk factors for conversion to THA. Another study (19) identified age > 40 years old and Tönnis grade 1 as risk factors for future conversion to THA.

The results of pre- and postoperative PRO's, graft choice, need for revision arthroscopy or conversion to THA for each study are summarized in Table 3.

**Table 3:** Graft choices, preoperative and postoperative functional scores, *p*-values, and reoperations after reconstruction.

**Study**	**Graft choice**	**Pre-op functional score** **Mean ± SD (range)**	**Post-op functional score** **Mean ± SD (range)**	***p*-value**	**Reoperations**
Lebus et al. (15)	ITB	mHHS: 65 (53–81) HOS-ADL: 71 (56–81) SF-12 PCS: 41.6 (33.9–48.1) SF-12 MCS: 55.0 (49.0–60.2) HOS-SS: 47 (28–66) WOMAC: 27.0 (15.5–40.5)	mHHS: 85 (63–92) HOS-ADL: 90 (76–96) SF-12 PCS: 53.1 (43.8–57.2) SF-12 MCS: 57.6 (52.8–59.6) HOS-SS: 75 (50–94) WOMAC: 9.0 (2.0–22.0) Satisfaction: 9 (6–10)	<0.01 <0.01 <0.01 0.311 <0.01 <0.01	42 THA 35 revisions
Amar et al. (16)	Indirect head of rectus femoris	mHHS: 67.1 (49.5–82.5)	mHHS: 97.8 (73.7–100)	<0.0001	No THA or revisions
Rathi and Mazek (17)	Indirect head of rectus femoris	mHHS: 56 (54–60)	mHHS: 93 (90–97) Satisfaction: 9.1 (8–10)	NR	No THA or revisions
Matsuda and Burchette (18)	Gracilis tendon	NAHS: 41.9 (25–64)	NAHS: 92.4 (83–99)	0.008	No THA or revisions
Nakashima et al. (19)	ITB	mHHS: 67.3 ± 14.9 NAHS: 63.0 ± 18.3	mHHS: 93.1 ± 11.9 NAHS: 88.2 ± 13.2	<0.001	3 THA 3 revisions
Locks et al. (20)	Indirect head of rectus femoris and capsule	HOS-ADL: 73 HOS-SS: 52 mHHS: 66	HOS-ADL: 89 HOS-SS: 79 mHHS: 89 Satisfaction: 9 (3–10)	<0.05 <0.05 <0.05	No THA or revisions
Maldonado et al. (21)	Semitendinosus tendon	mHHS: 65.8 ± 19.9 (29–96) NAHS: 58.5 ± 13.3 (35–79) HOS-SS: 40.1 ± 18.2(19–78) VAS for pain: 5.9 ± 2.1 (2–9)	mHHS: 81.4 ± 16.1 (57–100) NAHS: 82.4 ± 15.6 (56–100) HOS-SS: 70.9 ± 26.2 (27–100) iHOT-12: 68.8 ± 24.7 (27–100) VAS for pain: 2.7 ± 2.0 (0–6) Satisfaction: 6.6 ± 3.3 (0–10)	NR	1 THA 1 revision

*ITB, iliotibial band; mHHS, modified Harris Hip Score; HOS-ADL, Hip Outcome Score—Activities of Daily Living; SF-12 PCS, 12-Item Short Form Health Survey Physical Component Summary; SF-12 MCS, 12-Item Short Form Health Survey Mental Component Summary; HOS-SS, Hip Outcome Score—Sport Subscale; WOMAC, Western Ontario and McMaster Universities Osteoarthritis Index; NAHS, Non-Arthritic Hip Score; VAS, Visual Analog Scale; THA, Total Hip Arthroplasty; NR, not reported*.

## Discussion

The main finding of this systematic review was that arthroscopic labral reconstruction with autografts results in consistently improved patient outcomes. Arthroscopic labral reconstruction with autografts has been demonstrated to be a reliable surgical procedure for patients that present with persistent pain and functional limitations in the hip due to labral pathologies not amenable to repair, such as complex tears, degenerative, previously debrided, ossified or hypoplastic labrum. Furthermore, autografts inflict minimal donor-site morbidity and avoid the inherent risks and costs of allografts.

The suction seal of the acetabular labrum plays an important role in hip kinematics, function, stability, and intra-articular fluid pressurization, which is important for the protection of the cartilage matrix and in decreasing friction between the femoral head and acetabular surfaces. A cadaveric study has shown that simulated conditions of labral tears or labral resection decrease the pressurization of intra-articular fluid, and that subsequent repair and/or reconstruction, significantly restores this pressurization (3). Another biomechanical study with cadaveric specimens demonstrated that labral tears and resection decrease the resistance to distraction, and that repair and reconstruction improved distractive stability of the hip fluid seal (4). These findings demonstrate the rationale for restoring labral function at the time of hip arthroscopy, either by repairing the labrum when possible, or reconstructing in situations where it cannot be primarily repaired, rather than debriding or resecting the labrum given that removed tissue does not regenerate.

A study by Miozzari et al. (35) has shown that there is no regrowth of any structure similar to labrum after excision down to bleeding bone, and that these patients present with worse outcomes following excision. For this reason, reconstruction of the labrum is recommended. A study by Ejnisman et al. (36) reported that revision arthroscopies to treat adhesions and residual chondral lesions following labral reconstructions demonstrated graft incorporation and maintenance of the suction seal. This corroborates the findings of Shi et al. (37) who reported fully filled labrum defects in six and partially filled defects in three of nine animals in a porcine model, 24 weeks after reconstruction of the defects with gluteus medius tendon.

The first article regarding labral reconstruction was published in 2009, by Sierra and Trousdale (38). In their series of five patients, they reconstructed the labrum with an autologous ligamentum teres capitis graft after surgical dislocation of the hip, demonstrating significant improvement in the UCLA Score, from 5 (range, 2–6) preoperatively to 8.2 (range, 6–10) postoperatively, with a minimum of 5 months of follow-up. Since then, several other studies have been published, reporting improved outcomes with labral reconstruction, including both open and arthroscopic techniques utilizing different autografts sources including ITB, indirect head of rectus femoris, gracilis and semitendinosus tendons and capsule tissue, and allografts, such as semitendinosus and anterior tibialis tendons and fascia lata tissue (14–21, 39, 40). Biomechanically, all of these demonstrate similar cyclic elongation behavior in response to simulated physiologic forces (20).

The results of this systematic review are corroborated by previous reviews, which have demonstrated labral reconstruction to be an effective treatment for irreparable labrum, with good outcomes in properly indicated cases (11, 13, 23, 24). Trivedi et al. (11) reported that the score change for mHHS in 10 of 11 studies included in their systematic review ranged from 11 to 36, with the minimal clinically important difference (MCID) being 8 (33, 34, 41). Rahl et al. (23) found a significant improvement (*p* < 0.001) in the mHHS of 29.0 points, including six studies in their systematic review and meta-analysis. However, those studies have included articles in which both auto- and allografts have been used, and using both open and arthroscopic techniques. Despite the current cadaveric donor screening and tissue processing methods, allografts have the theoretical risk of disease transmission, with reported cases of HIV, Hepatitis B and C infections (likely due to window periods or human error), and *Clostridium* septic arthritis after their use (27–29). In addition, delayed incorporation, limited availability, and increased costs are also considerable disadvantages for allografts (21, 23, 27, 29). Open procedures with surgical dislocation of the hip have been demonstrated to present with increased incidence of reoperations (25) and slower recovery and return to sports (26). Given this, the authors focused this systematic review, and only included studies describing autograft-based labral reconstructions utilizing arthroscopic technique.

When compared to other techniques to treat labral tears not amenable to repair, labral reconstruction with autografts has shown superior outcomes compared to simple debridement. Domb et al. (22) reported significant differences in the mean changes for NAHS and HOS-ADL of 24.8 and 21.7, respectively for patients undergoing labral reconstruction with a gracilis tendon autograft, and 12.5 and 9.5 for patients undergoing labral debridement, with no differences in functional outcomes and complications compared to labral reconstruction with allografts as shown in the recent Systematic Review by Rahl et al. (23). Therefore, when treating patients presenting with labral insufficiency, debridement alone should be avoided, and reconstruction with either allo- or autografts should be performed. As there is no evidence to support one type of graft over the other, patient and surgeon preferences, and the availability of allografts will guide this choice.

Only one of the seven studies included in this systematic review has reported complications related to the harvesting of autografts, which was pain at the donor site following gracilis tendon harvesting (18). However, this may be an underrepresentation of hamstring harvest donor site morbidity, given that larger series involving anterior cruciate ligament reconstructions have reported a higher incidence of donor site morbidity, such as saphenous nerve injury, weakness of knee flexion, and hypoesthesia (42, 43). For other autografts such as ITB, Philippon et al. (44) pointed out that infection, pain, and muscular hernia are potential complications. For this reason, considering a local graft tissue for labral reconstruction, such as the indirect head of rectus femoris or hip capsule, may be a good option. These grafts can be harvested through arthroscopic portals, avoiding the necessity for additional incisions. In the present review, two articles have studied the use of the indirect head of rectus femoris (16, 17), and one has studied both the indirect head of rectus femoris and capsule tissue (20) as grafts for labral reconstruction. All three studies reported significant improvements in the mHHS, from 67.1, 56, and 66 preoperatively, to 97.8, 93, and 89 postoperatively, respectively in the studies by Amar et al. (16), Rathi and Mazek (17), and Locks et al. (20), with no complications, need for revision arthroscopy, or conversion to THA in the follow-up period. The authors highlight the advantages of using a local graft, which include no donor-site morbidity, no use of a cadaveric graft, preservation of the distal blood supply of the grafted tissue, and elimination of the need for back table work and an additional incision (16, 17, 20). There is not a consensus on the best source of autograft or in which cases a specific type of autograft is better indicated. However, for surgeons who prefer to use local grafts, due to their inherent advantages, Locks et al. (20) has suggested using capsule tissue for labral deficiencies located between 12 and 9 o'clock, and indirect head of rectus femoris for defects between 12 and 3 o'clock. The authors cautioned that these grafts should be used for segmental reconstructions, and are not good options for defects larger than 1 cm or for patients with LCEA < 25°, as it could lead to hip microinstability. For such cases, ITB or gracilis or semitendinosus tendon autografts are recommended, taking into consideration that hamstrings harvesting may cause incisional pain and weakness for knee flexion.

The limitations of this systematic review include the level of evidence of the retrospective level III and IV studies included, which were also limited to short and mid-term outcomes of relatively small patient sample sizes. Prospective and randomized trials, with longer follow-up and larger sample sizes would be preferred for a more comprehensive and reliable assessment of arthroscopic labral reconstructions. Such design, including randomization of graft choice, would permit the comparison of different graft choices and their impact on outcomes and donor site morbidity. Specifically, the most interesting comparisons would be hamstring and ITB grafts with grafts such as the indirect head of rectus femoris tendon that may be harvested without causing donor-site complications.

## Conclusion

The acetabular labrum plays a key role in hip kinematics, function, and stability. In cases of labral insufficiency or other conditions not amenable to repair, arthroscopic acetabular labrum reconstruction with autografts results in significant improvement in short- and mid-term patients reported outcomes, in young patients without moderate or advanced osteoarthritis of the hip.

## Data Availability Statement

All datasets generated for this study are included in the article/ supplementary material.

## Author Contributions

FB: search strategy, screening of studies, data extraction, manuscript writing, and final revision. BW: manuscript writing and final revision. EP: statistical analysis, plot preparation, and final revision. MN: search strategy, manuscript writing, and final revision. FG: screening of studies, data extraction, and final revision. GL: search strategy and final revision. LM: search strategy and final revision. JC: manuscript writing and final revision.

## Conflict of Interest

The authors declare that the research was conducted in the absence of any commercial or financial relationships that could be construed as a potential conflict of interest.
